# Immunotherapy Combined with Radiation in Malignant Melanoma without BRAF Mutations Brain Metastases—Favorable Response after Immunotherapy Continued beyond Progression

**DOI:** 10.3390/jpm14010086

**Published:** 2024-01-12

**Authors:** Roxana-Andreea Rahnea-Nita, Laura-Florentina Rebegea, Radu-Valeriu Toma, Horia Mocanu, Ioana Soare, Raul Mihailov, Alexandru Nechifor, Mădălin Guliciuc, Georgiana Bianca Constantin, Gabriela Rahnea-Nita

**Affiliations:** 1 The Clinical Department, The Faculty of Medicine, The University of Medicine and Pharmacy “Carol Davila”, 050474 Bucharest, Romania; roxana.rahnea-nita@umfcd.ro (R.-A.R.-N.); radu.toma@umfcd.ro (R.-V.T.); 2 The Oncology-Palliative Care Department, “Sf. Luca” Chronic Disease Hospital, 041915 Bucharest, Romania; gabriela.rahnea-nita@umfcd.ro; 3 The Clinical Department, The Faculty of Medicine and Pharmacy, “Dunarea de Jos” University in Galati, 800008 Galati, Romania; laura.rebegea@ugal.ro (L.-F.R.); raul.mihailov@ugal.ro (R.M.); alexandru.nechifor@ugal.ro (A.N.); madalin.guliciuc@ugal.ro (M.G.); 4 The Radiotherapy Department, “Sf. Ap. Andrei” County Emergency Clinical Hospital, 800579 Galati, Romania; 5 The Research Center in the Field of Medical and Pharmaceutical Sciences, ReFORM-UDJ, 800010 Galati, Romania; 6 The Radiotherapy Department, The Oncological Institute, 022328 Bucharest, Romania; 7 The Clinical Department, The Faculty of Medicine, “Titu Maiorescu” University, 040051 Bucharest, Romania; horia.mocanu@prof.utm.ro (H.M.); ioana.soare@prof.utm.ro (I.S.); 8 The E.N.T Department, Gaesti City Hospital, 135200 Gaesti, Romania; 9 The Urology Department, “Sf. Ap. Andrei” County Emergency Clinical Hospital, 800579 Galati, Romania; 10 The Morphological and Functional Sciences Department, The Faculty of Medicine and Pharmacy, “Dunarea de Jos” University in Galati, 800008 Galati, Romania; 11 The Clinical Department, The Faculty of Midwifery and Nursing, The University of Medicine and Pharmacy “Carol Davila”, 050474 Bucharest, Romania

**Keywords:** malignant melanoma without BRAF V 600 mutations, brain metastases, radiotherapy, immunotherapy beyond progression, favorable response

## Abstract

We present the case of a patient who was diagnosed in 2018 with nodular Malignant Melanoma (MM) without BRAF V 600 mutations stage 3 C (pT4b pN1a M0), and who underwent adjuvant citokines treatment with Interferon alpha 2b-48 weeks. Immunotherapy was initiated in January 2021 for lung and lymph node metastases. In June 2021, there was a partial response of the lung and lymph node metastases, but there was also progression to brain metastases. Immunotherapy was continued and Whole Brain Radiotherapy (WBRT) was performed. In September 2023, the imaging investigations revealed a favorable response, with no lesions suggestive of secondary determinations. The combination of Radiotherapy (RT) and Immunotherapy (IT) with Immune Checkpoint Inhibitors (ICI) has an abscopal effect. There is a coordinated action in the combination of RT and IT in order to obtain a common result, with the antitumor effect being greater than if RT or IT acted separately.

## 1. Background

Malignant melanoma is an aggressive type of cancer. The most common subtypes of cutaneous malignant melanoma are superficial spreading melanoma, nodular melanoma, lentiginous melanoma, and acral melanoma.

Pathological aspects of the primary melanoma, such as tumor thickness, the rate of mitosis, and the presence of ulceration, are major prognostic factors. Immunohistochemical staining for molecular markers are important in staging and in prognosis assessment.

The most common molecular alteration in patients with metastatic melanoma are mutations in the BRAF gene, in particular BRAF V600 (BRAF V600 E, BRAF V 600 K). Mutation testing for actionable mutations is mandatory in patients with resectable or unresectable stage III or stage IV.

Brain metastases were reported in 28.2% of the patients with malignant melanoma, the prognosis of these patients being unfavorable.

The therapeutic options for brain metastases include surgical resection, whole-brain radiation therapy (WBRT), stereotactic radiosurgery (SRS), combinations between WBRT and SRS, and immunotherapy with Immune Checkpoint Inhibitors (ICI) [1,2].

There is a significant improvement in the overall survival of patients with melanoma brain metastasis in the era of novel therapies.

As a systemic treatment, immunotherapy is the primary option for malignant melanoma patients with brain metastases in the absence of the V600 mutation of the BRAF gene, being a safe therapeutic option [3].

Single-agent programmed death receptor-1 (PD-1) antibodies (Nivolumab, Pembrolizumab), the combination of PD-1 antibodies + cytotoxic T-lymphocyte–associated antigen 4 (CTLA-4) (Ipilimumab), and the monoclonal antibody against PD-L1 (Atezolizumab) induce durable response rates [4,5].

Radiotherapy (RT) may cause a systemic immune response that can enhance the response to Immune Checkpoint Inhibitors. At the same time, immunotherapy may increase the sensitivity of the tumor to radiotherapy, thus being a synergistic action of RT and immunotherapy, in order to obtain a common result, the antitumor effect being greater than if radiotherapy and immunotherapy acted separately [6,7,8].

The combination of RT and immunotherapy with ICI (antibody therapies against CTLA-4, PD-1) has an abscopal effect, respectively; localized radiation has the ability to induce an antitumor response at distant sites that were not subjected to targeted RT [7,9,10].

Further studies are necessary to characterize the timing and the optimal sequence.

The results regarding the enhancement in abscopal activity by the combination of RT with immunotherapy are variable. Further research towards improvement in abscopal activity is warranted [9].

The prognosis of patients with malignant melanoma varies and it is related to the stage of the disease at the diagnosis. Melanomas without BRAF mutations tend to be less aggressive than BRAF mutation melanomas. Important prognostic features of invasive melanomas include Breslow thickness, ulceration, and the mitotic rate. In order to optimize the patient’s care, both accurate diagnosis and staging (via pathological examination and imaging) and treatment planning are important. A multidisciplinary team approach is considered the standard of care for the treatment of a variety of cancers, including malignant melanoma, and it involves an active collaboration of doctors from different specialties (pathological anatomy, dermatology, plastic surgery, medical oncology, radiation therapy, radiology) during the evolution of the disease. Specialists’ perspectives lead to better results, increased quality of life and patient survival, as well as increased patient satisfaction [11,12,13,14,15,16,17].

Due to the complexity of novel therapies, it is important for physicians to update their knowledge in the management of metastatic malignant melanoma.

## 2. Case Presentation

A 62-year-old male patient presented to the Oncology-Palliative Care Department at St. Luke Hospital in Bucharest in January 2021, with the diagnosis of nodular malignant melanoma to the upper back, without BRAF V 600 mutation.

The malignant melanoma was discovered accidentally during a routine examination in June 2018, and the patient did not know of its existence.

After the surgical resection in June 2018, the pathological staging was pT4b pNx M0.

The histopathological examination revealed the following: invasive nodular melanoma; the maximum thickness (Bres-low) of the tumor—19 mm; ulceration present; microsatellites—unidentified; peripheral margin—free of invasive melanoma; distance from the invasive melanoma to the nearest peripheral margin (millimeters)—3 mm; deep margin—free of invasive melanoma; distance from the invasive melanoma to the deep margin (millimeters)—10 mm; mitotic rate—5 mitoses/mm^2^; and (Clark) anatomical level of invasion—V(melanoma invades the dermis). Lymphovascular invasion, intratumoral lymphocytic infiltrate, and tumor regrowth were not identified.

Immunohistochemical examination: S100-diffuse positive, HMB 45-positive, Ki67-positive in tumor cells dispersed throughout the thickness of the tumor, AE1/AE3-negative.

The lymphatic scan examination in July 2018 revealed sentinel lymph nodes in the left axillary area and at the level of the left postero-lateral chest wall.

After the lymph node dissection of the sentinel lymph node in July 2018, the patient was evaluated as stage 3 C (pT4b pN1a M0).

Histopathological exam and immunophenotyping of the sentinel lymph node:

- Sentinel lymph node 1 (left axillary region): melanoma metastase, S 100-positive, Melan A(Mart-1)-positive.
- Sentinel lymph node (scapulary region): negative for metastase, S 100-negative, Melan A(Mart-1)-negative.

The patient has an Eastern Cooperative Oncology Group Performance Status (ECOG) = 1.

The patient does not have a family history of melanoma. He worked as a turner. Regarding the comorbidities, he has type 2 diabetes, treated with insulin.

Previously, the patient had undergone adjuvant cytokines treatment with Interferon alpha 2b, for 48 weeks in another medical facility.

The computed tomography (CT) in March 2020 revealed four pulmonary nodes described as metastases, and in May 2020, the CT investigation revealed no secondary cerebral determinations. The pulmonary nodes (four) with the appearance of secondary determinations seemed slightly increased in dimension compared to the examination in March 2020. The occurrence of new suspicious nodes was not detected. The two infracentimetric liver lesions with a cystic appearance had an unmodified appearance upon the CT scan.

Subsequently, a CT scan of the head, chest, abdomen, and pelvis with contrast medium was performed in December 2020, which revealed no CT signs of local-regional tumor relapse. Secondary pulmonary determination in numerical and dimensional progression. Newly emerged mediastinal and left pulmonary hilum tumoral adenopathies. No secondary cerebral, hepatic, nor bone determinations.

The patient was admitted to St. Luke Hospital, the Chronic Oncology-Palliative Care Department in January 2021, with a performance status ECOG = 1, and immunotherapy with Nivolumab was initiated, 240 mg every 14 days.

The CT of the head, thorax, abdomen, and pelvis with contrast medium performed in June 2021 revealed the occurrence of two nodular brain lesions suspicious for secondary determinations—to be completed with a brain Magnetic Resonance Imaging (MRI) examination. Numerical and dimensional regression of secondary pulmonary determinations and mediastinal adenopathies.

**Figure 1:**
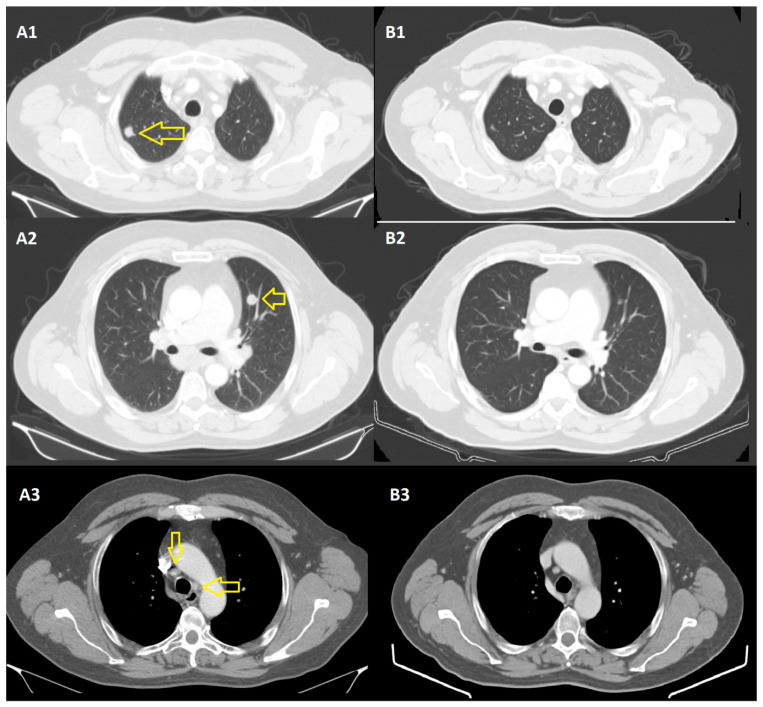
CT scans from December 2020 (**A1**–**A3**), compared to those from June 2021 (images (**B1**–**B3**)), where pulmonary metastases (yellow arrows) and adenopathies are in regression.

Thus, we noticed a partial remission of the secondary lung and lymph node determinations 6 months after the initiation of immunotherapy (Figure 1).

**Figure 2:**
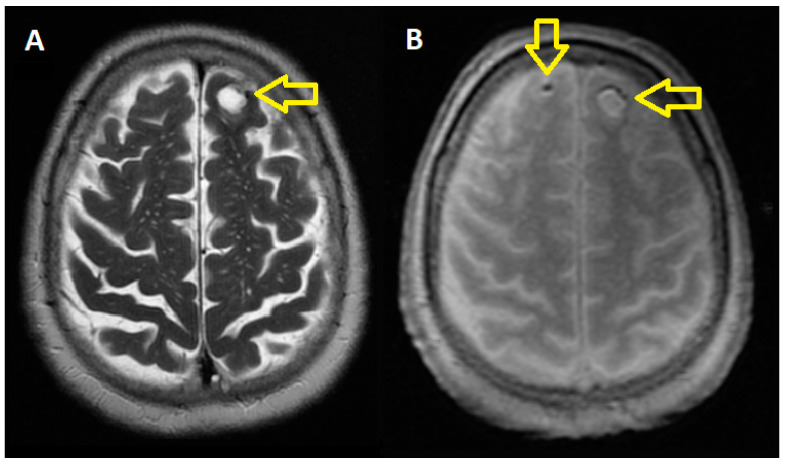
Brain Magnetic Resonance Imaging (July 2021): On the T2 MRI sequence (**A**) and the T1 MRI sequence (**B**), the image reveals a prominent subcortical frontal lesion on the left side, which likely contains melanin and appears to be a secondary determination. Additionally, there are seven small, micro-hemorrhagic lesions found in the cortical-intergyral regions of the frontal lobes, bilaterally, as well as in the right posterior parietal region and at the level of the right thalamus. These lesions may be indicative of either cortical micro-hemorrhages in a hypertensive context or micro-secondary hemorrhagic determinations. Yellow arrows: Subcortical lesion- the left image and micro-hemorrhagic lesions in the right image.

The cranio-cerebral MRI performed in July 2021 revealed a high subcortical frontal nodular lesion on the left side (most likely with melanin content), with an MRI appearance corresponding most likely to a secondary determination. Another seven micro-nodular micro-hemorrhagic cortical-intergyral frontal high bilateral lesions, right posterior parietal, and at the level of the right thalamus corresponded either to cortical micro-hemorrhages in a hypertensive context or to micro-secondary hemorrhagic determinations (Figure 2). It should be mentioned that the patient did not have any neurological signs.

The multidisciplinary therapeutic indication board recommended Whole Brain Radiotherapy, which was carried out in August 2021, through the administration of 30 Gy/10 fractions (3 Gy/fraction).

Next, after the completion of radiotherapy, the patient had a performance status ECOG = 1.

**Figure 3:**
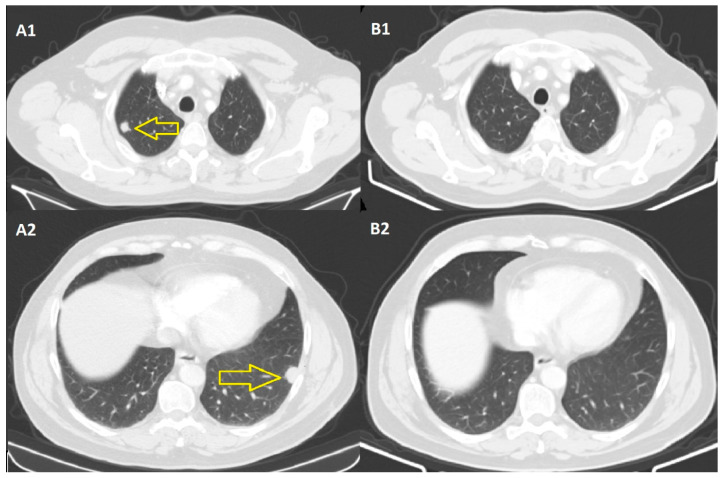
CT scans from December 2020 (**A1**,**A2**), where pulmonary metastases (yellow arrows) were evident on the CT images, compared to the CT scans from September 2021 (**B1**,**B2**), which indicate normal limits.

In September 2021, a CT of the thorax, abdomen, and pelvis was performed, which revealed an oncological aspect within normal limits (Figure 3).

Thus, we are witnessing a complete remission of secondary pulmonary and lymph node determinations, 9 months after the initiation of immunotherapy, and the observation concerns the abscopal effect that most probably radiotherapy contributed to this result.

The multidisciplinary board for therapeutic indication recommended the continuation of the treatment with Nivolumab in September 2021.

**Figure 4:**
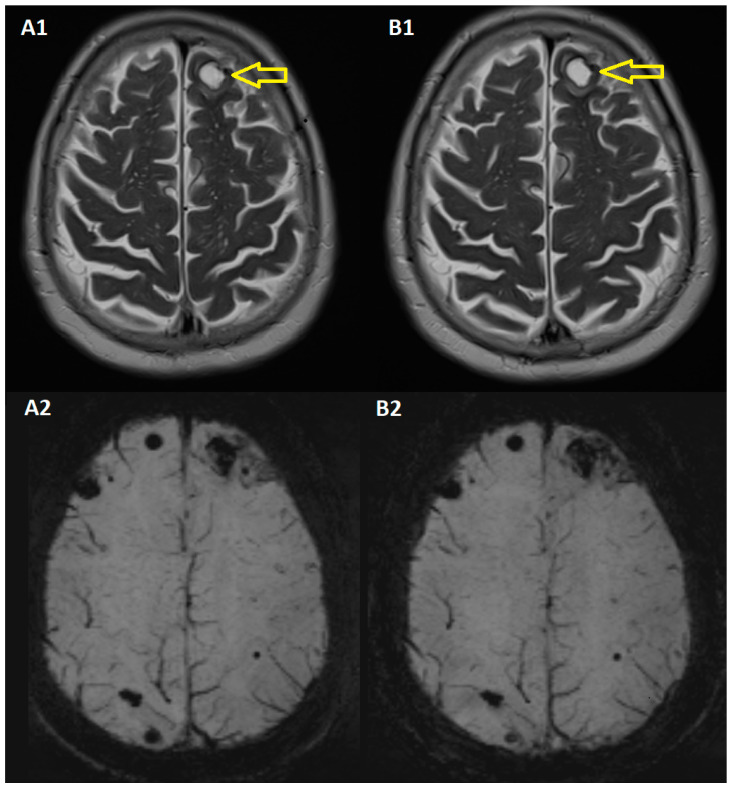
Brain Magnetic Resonance Imaging in October 2021 T2 (**A1**), SWI (**A2**) vs. December 2021 T2 (**B1**), SWI (**B2**): In the MRI from October 2021 (images (**A1**,**A2**)), the frontal lesion is noticeable and multiple lesions are observed, primarily on the brain’s convexity, including both gyral and cortical-subcortical junction lesions. In the MRI from December 2021 (images (**B1**,**B2**)) were similar lesions. Yellow arrows: Subcortical lesion.

In October 2021, an MRI examination of the skull was performed. Compared to the examination in July 2021, the following were revealed: “the frontal subcortical lesion was found high on the left side, currently without obvious gadophilia, maintaining the diffusion restriction, most likely due to the hemorrhagic content, in minimal regression; the supratentorial cerebral nodular lesions described in the previous examination, with the increase of the area on a susceptibility sequence, without a corresponding hyper-signal on the diffusion side and not influenced by the administration of the contrast medium, e.g., currently measuring 7 mm high right frontal (from 4 mm), 10 mm left frontal (from 6 mm), 9 mm right posterior parietal (from 5 mm), 7 mm right capsular-thalamic (from 3 mm); without cortico-subcortical areas suspicious for a recent ischemic vascular substrate” (Figure 4).

In November 2021, the multidisciplinary board recommended:

- Consultation for Stereotactic radiosurgery (SRS).
- Continuation of the treatment with Nivolumab.

The SRS consultations in November 2021 and January 2022 decided that the evolution after WBRT was favorable and recommended just MRI monitoring.

The MRI examination in December 2021, compared to the one in October 2021, revealed the following conclusions: secondary supratentorial brain lesions with numerical and dimensional similarity compared to the examination in October 2021.

In April 2022, a CT scan of the thorax, abdomen, and pelvis was performed, which revealed a normal oncological aspect, similar to the examination in September 2021.

The CT scan (brain) performed in May 2022 revealed secondary supratentorial cerebral lesions with a SWI a-signal without gadolinophilia—numerically and dimensionally similar to the examination in December 2021. No new lesions were detected.

The CT scan (brain, thorax, abdomen, and pelvis) in October 2022 was similar to the ones of the previous examination.

Therefore, a complete remission of the secondary pulmonary and lymph node determinations was maintained, the duration of this complete remission being now of 13 months.

The board for therapeutic indications reunited in May 2022 and recommended the continuation of the treatment with Nivolumab.

The MRI examination performed in October 2022 revealed secondary supratentorial cerebral lesions, numerically and dimensionally similar to the examination in May 2022. No new lesions.

At the cerebral level, the disease was stationary in October 2022, compared to December 2021 and May 2022.

In September 2023, a CT scan of the head, thorax, abdomen, and pelvis was performed with contrast medium, and the interpretation was compared to the CT scan of the thorax, abdomen, and pelvis performed in October 2022.

The CT examination in September 2023 in the cerebral and thoraco-abdomino-pelvic areas performed with and without contrast medium, was interpreted comparatively with the CT scan of the thorax, abdomen, and pelvis performed in October 2022, and revealed:

### 2.1. At the Cerebral Level

“No hypondense areas suggestive of a recently formed ischemic substrate visible on the CT scan at the time of the examination.

No intra- or pericerebral hemorrhagic accumulations.

Hypodensities in the fronto-parietal white matter bilaterally, with a microangiopathic substrate.

**Figure 5:**
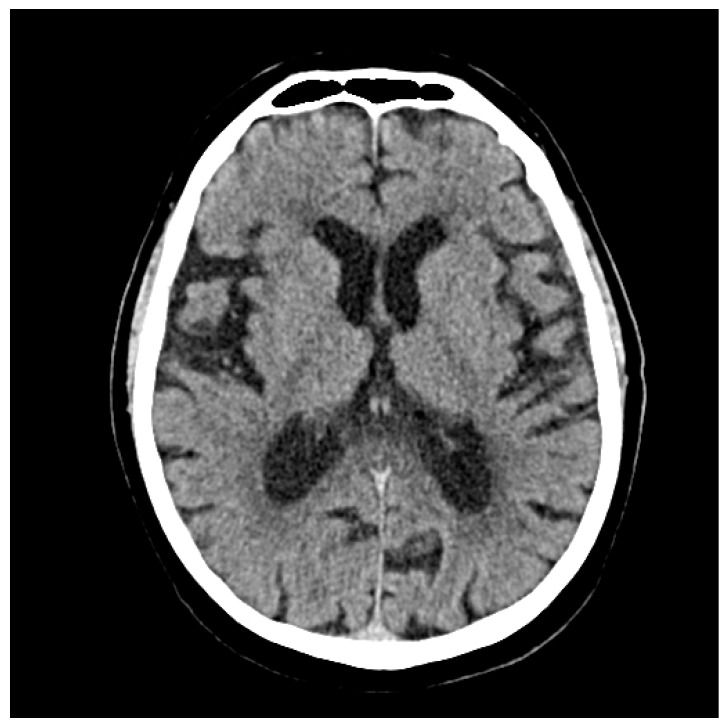
The computed tomography of the brain (September 2023): no acute vascular lesions, no expansive processes visible on the CT scan at the time of the examination.

Conclusions:No acute vascular lesions, no expansive processes visible on the CT scan at the time of the examination” (Figure 5).

### 2.2. At the Thoracic Level

No fluid nor gas effusions in the pleuro-pericardial space.

No lung consolidation processes and no suspicious nodular images at the lung parenchyma level.

No suspicious adenomegaly.

### 2.3. At the Abdominal-Pelvic Level

‘’Liver with normal dimensions (cranio-caudal LDH diameter of 14.3 cm), clear regular margins, without focal processes indicative of secondary determinations.

No free fluid nor confined at the abdominal-pelvic level. No abdominal-pelvic adenomegaly.

No focal lesions at the level of the scanned bone segments, suspicious in an oncological context’’.

**Figure 6:**
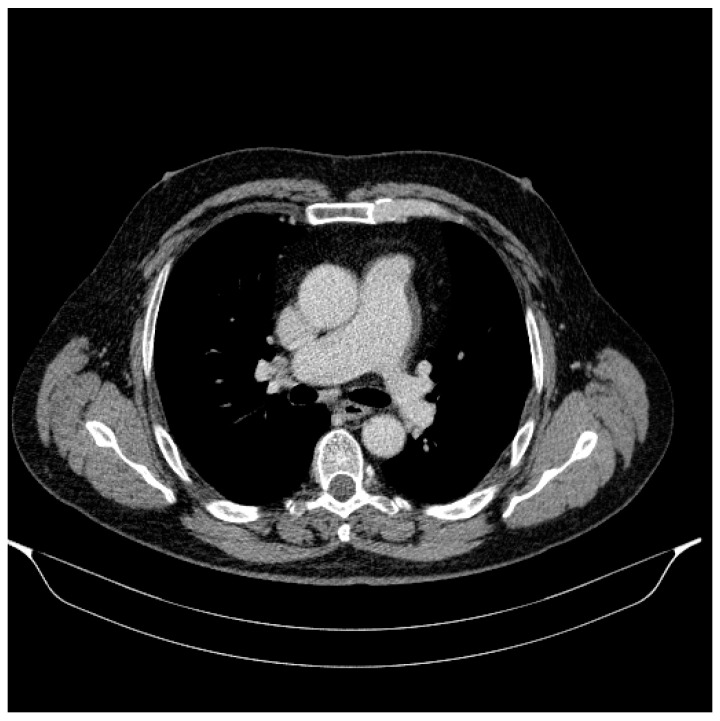
The computed tomography of the chest (September 2023): no lesions with a suggestive appearance for secondary thoracic determinations.

Conclusions: “No lesions with a suggestive appearance for secondary determinations at the thoraco-abdomino-pelvic level” (Figure 6).

Therefore, next, in September 2023, the complete remission of the secondary pulmonary and lymph nodes is maintained (the duration of the complete remission being 24 months: September 2021–September 2023). Moreover, the complete remission of cerebral secondary determinations is also noticed, compared to October 2022.

In conclusion, the disease is in complete remission in September 2023.

In November 2023, the patient continues the maintenance treatment with Nivolumab, having an ECOG performance status = 1.

## 3. Discussion

The main objective of this quick review is to identify the relevant data regarding the current treatment of melanoma brain metastases; we have summarized the recent knowledge arising in the context of novel therapies.

The treatment options for melanoma brain metastases include:

### 3.1. Surgery

Surgery should be considered for patients with symptomatic and large brain metastases.

### 3.2. Stereotactic Radiosurgery and Whole-Brain Radiation Therapy

SRS is considered for patients with 1 to 4 brain lesions. SRS can be used as an alternative to surgery for lesions that are not larger than 3 cm in diameter and depth.

WBRT is used for the following:

1. A palliative purpose for patients who are not eligible for systemic therapy.

Thompson JF et al., in a systematic review performed in 2022, revealed that from the time of diagnosis of brain metastasis, the median survival after SRS alone was 7.5 months [18].

The same review revealed that from the time of diagnosis of brain metastasis, the median survival after WBRT alone was 3.5 months [18].

Given the proved efficacy of the new systemic therapies, radiotherapy as a single treatment method no longer plays a role in the modern treatment of melanoma brain metastases.

2. WBRT is recommended for patients with brain metastases who make progress during systemic therapy and who are not eligible for surgery or SRS [19].

### 3.3. Immunotherapy

Many studies have shown that combination therapy (anti-CTLA-4 + anti-PD-1) increases the response rates compared to monotherapy [3,4].

Some studies did not reveal differences between the survival of patients with negative BRAF malignant melanoma with brain metastases who underwent double immunotherapy (CTLA-4 + PD-1), compared to those who underwent immunotherapy with one agent PD-1 [20].

The results of three randomized controlled trials suggest that Ipilimumab, Nivolumab, and Pembrolizumab, as a monotherapy, and the combination therapy of Nivolumab plus Ipilimumab can improve or maintain the quality of life, being well tolerated [21,22,23,24].

The life quality of patients with metastatic cancer is related to the oncological treatment performed, but also to the adaptation mechanisms of the patients with the disease [25,26,27,28,29].

The systemic activity of Immune Checkpoint Inhibitors is also associated with adverse events, namely, peripheral neuropathies, encephalitis, and paraneoplastic disorders that affect the central nervous system. These adverse reactions have an incidence of less than 5%, but the complications are severe and can lead to long-term disabilities, even death. Their management consists in the interruption of immunotherapy and the administration of immunomodulatory therapies [30,31,32,33,34,35].

Although there are remarkable data regarding the overall survival in patients with advanced malignant melanoma under treatment with anti-PD1-based immunotherapy, the optimal duration of the treatment is not yet known [36].

### 3.4. Stereotactic Radiosurgery with Immunotherapy

Minniti G. et al. [37]., in a study conducted on 80 patients treated with SRS followed by immunotherapy with Ipilimumab or Nivolumab, found that 78% of the patients treated with Nivolumab were alive after 12 months, while the percentage of the patients treated with Ipilimumab who were alive after 12 months was 68% [37].

In another study, conducted by Gaudy-Marqueste C. et al., the median overall survival in BRAF wild-type patients treated with immunotherapy after SRS was 12.3 months [38].

Diaz, M.J. et al., in a systematic review carried out in 2023, reveal that the current treatment of brain metastases in malignant melanomas is directed towards the combination of stereotactic radiosurgery (SRS) and immunotherapeutic agents [39].

Wilson T.G. et al., in a retrospective study on 70 patients that was carried out between 2014 and 2020, in which the treatment consisted of surgery alone/surgery + SRS/SRS alone, followed by dual immunotherapy (Ipilimumab + Nivolumab)/single agent immunotherapy (Ipilimumab/Nivolumab/Pembrolizumab), the patients who were treated with Ipilimumab had a median overall survival of 14.3 months, and the patients who were treated with anti-PD-1 therapy, either Nivolumab or Pembrolizumab, had a median overall survival of 14.1 months, and the patients who were treated with dual immunotherapy, the combination of Ipilimumab and Nivolumab, had a median overall survival of 26.7 months [40].

Porte. J et al., in a retrospective study performed on patients with non-small lung cancer with brain metastases, reveal that the concurrent administration of SRS and IT provided better local and regional control compared to SRS administered before or after IT, and the concomitant treatment did not induce more acute neurologic toxicity [41].

### 3.5. Whole-Brain Radiation Therapy with Immunotherapy

Immunotherapy administered concomitantly with radiotherapy highlights the potential synergism of the two therapeutic methods [42,43,44,45,46,47,48].

Glitza Oliva I.C. et al., in a retrospective study conducted on 70 patients treated between 2005 and 2012 which compared the effectiveness of SRS/WBRT to which Ipilimumab was either added or not, revealed that the addition of Ipilimumab to SRS radiation improved survival compared to SRS alone, while the addition of WBRT did not significantly improve survival in the WBRT subset [49].

Vosoughi E et al. conducted a retrospective study in California between January 2011 and June 2015, in which 79 patients with malignant melanoma and brain metastases were included, and the data regarding the overall survival were the following:

- The median overall survival from the time of diagnosis of brain metastases was 12.8 months.
- The median overall survival from the time of diagnosis of malignant melanoma was 60.5 months.
- The median overall survival from the time of the first whole brain radiation therapy was 6.8 months.
- The OS from the time of the initial brain metastasis in patients who were treated with anti-PD-1 antibody therapy before the initial brain metastasis was 8.5 months [50].

Comparatively, regarding our patient (Table 1):

- The overall survival from the time of diagnosis of brain metastases is 29 months; in November 2023, the patient underwent a complete remission of brain metastases
- The overall survival from the time of diagnosis of malignant melanoma is 65 months, with the patient being in complete remission in September 2023.
- The overall survival from the time of the first whole brain radiation therapy is 26 months.
- The overall survival from the time of initial brain metastasis in our patient who was treated with anti-PD-1 antibody therapy before the initial brain metastasis is 29 months.
- It is worth mentioning that in our patient’s case, the overall survival from the time of diagnosis of lung metastases is 44 months, and the durable complete response of lung metastases is 24 months in September 2023.

## 4. Conclusions

1. The complete remission of pulmonary and lymph node metastases, 9 months after the initiation of immunotherapy, is a durable remission, with a duration of 24 months, in September 2023.
2. Brain metastases, which appeared under the immunotherapeutic treatment, were treated with WBRT, followed by the continuation of immunotherapy, due to the clinical benefit and the good performance status.
3. A survival period of 29 months from the time of occurrence of treated brain metastases.
4. After an initial slight progression at the cerebral level, after radiotherapy, and under immunotherapy, the disease became stable at the cerebral level, and 28 months after the diagnosis of cerebral metastases, in September 2023, a complete remission was recorded.
5. The decision to continue immunotherapy after the occurrence of brain metastases was beneficial to the patient, who enjoyed a very good life quality, 29 months after their diagnosis.
6. In September 2023, the complete remission of the disease is recorded, 64 months after the initial diagnosis, respectively, 33 months after the initiation of immunotherapy, a treatment which was well tolerated, with no adverse effects.
7. The complete remission of the disease continues 24 months after the performance of WBRT, thus revealing the synergistic effect of the combination between immunotherapy and radiotherapy.
8. WBRT still has its role in combination with immunotherapy in the overall survival of patients with malignant melanoma brain metastases, through the synergistic action of the two therapeutic methods.
9. Further studies towards enhancement in abscopal activity are necessary.

## Figures and Tables

**Table 1:** Data on survival [50].

	Vosoughi E et al., Study between January 2011 and June 2015—79 Patients [50]	Our Patient (Overall Survival) June 2018—Alive in November 2023
Median overall survival from the time of diagnosis of brain metastases	12.8 months	29 months
Median overall survival from the time of diagnosis of malignant melanoma	60.5 months	65 months
Median overall survival from the time of the first whole brain radiation therapy	6.8 months	26 months
Median overall survival from the time of the initial brain metastasis in patients who were treated with anti-PD-1 antibody therapy before the initial brain metastasis	8.5 months	29 months

## Data Availability

Data are contained within the article.
